# Laser-induced voltage of table salt for deep ultraviolet pulsed laser detection

**DOI:** 10.1016/j.isci.2024.109424

**Published:** 2024-03-05

**Authors:** Xuecong Liu, Kun Zhao, Xinyang Miao

**Affiliations:** 1 College of Information Science and Engineering/College of Artificial Intelligence, China University of Petroleum, Beijing 102249, China; 2 College of New Energy and Materials, China University of Petroleum, Beijing 102249, China; 3 Beijing Key Laboratory of Optical Detection Technology for Oil and Gas, China University of Petroleum, Beijing 102249, China; 4 Key Laboratory of Oil and Gas Terahertz Spectroscopy and Photoelectric Detection, Petroleum and Chemical Industry Federation, China University of Petroleum, Beijing 102249, China

**Keywords:** Natural sciences, Physics, Applied sciences

## Abstract

To meet the requirements of fast response and simple process of deep ultraviolet (UV) pulsed laser detector, table salt (TS) was used as laser detection material in combination with a variable resistor to achieve single-pulse laser detection. Under the irradiation of a KrF excimer laser, the laser-induced voltage (LIV) of TS was influenced by the dynamic process of laser-induced plasma, and the whole process was well fitted with the sum of the three exponential functions. As the applied bias voltage (V_b_) and incident laser energy (E_in_) increased, the LIV amplitude (V_p_) increased and the response time decreased. When the variable resistor (R) was reduced to 14.7 Ω, the response time of LIV decreased from ∼300 μs to ∼20 ns, which is the same as the duration of laser pulse. This research provided a simple, low-cost, and fast method for the detection of UV single-pulse laser based on the laser-TS interaction.

## Highlights

• Table salt (TS) can be used as a material for UV single-pulse laser detection
• LIV response can characterize the dynamic process of laser-induced plasma
• The impedance effect of LIV can monitor UV laser pulse in real-time
• The logarithm of LIV peak is linearly correlated to laser energy

## Graphical abstract

**Figure:**
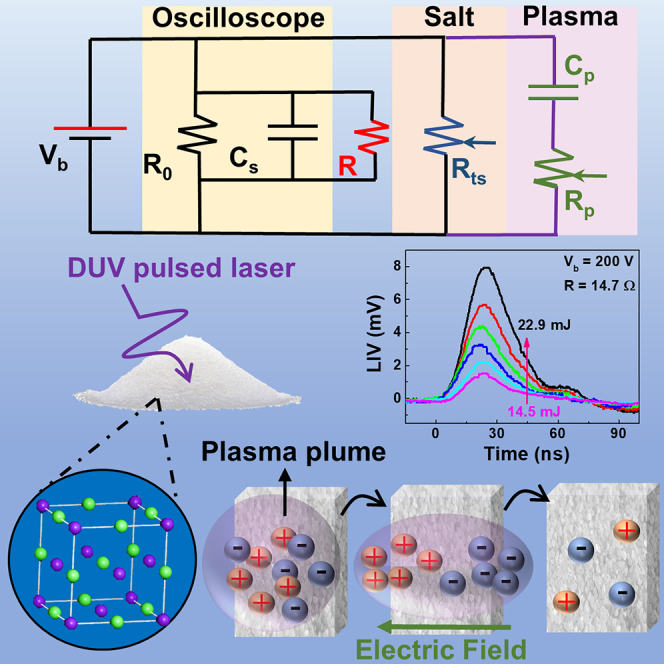

## Introduction

The high energy laser can lead to a rapid expanding hot and partially ionized vapor plume generation under the irradiation on the surface of the target material, which has important research value and significance in the field of precision micromachining, diagnostic techniques, medicine, material processing, nanoscale synthesis, etc.^1^^,^^2^^,^^3^^,^^4^^,^^5^ Since optical, acoustical, and electromagnetic phenomena, reflecting the sample characteristics, are generated after laser-induced plasma (LIP), quantitative and qualitative analysis of the sample can be achieved by these phenomena.^6^^,^^7^^,^^8^^,^^9^^,^^10^ It is crucial to measure the characteristics of the pulsed laser, especially for the real-time detection of single-pulse laser energy. With the development of laser measurement process, thermopile energy system, and wide-gap semiconductor detection materials are widely applied.^11^^,^^12^ In recent years, photodetectors have attracted extensive attention for ultraviolet (UV) laser detection due to its good detection performance.^13^ However, the high-energy UV pulse laser causes a challenge to traditional laser energy detection system due to its low thermal effect and narrow pulse width. Because the characteristics of LIP are affected by single-pulse laser energy, LIP is expected to be a sharp edge for high single-pulse laser detection.^14^ However, the plasma characteristics are also influenced by many factors such as the laser wavelength, the pulse duration, the target characteristics.^15^ It is more challenging to achieve rapid single-pulse laser signal acquisition and energy extraction based on laser-material interactions.

Because the plasma generated by laser irradiation can be used as a signal source and the electrical signal of the plasma can be collected by an external circuit, it is receiving more and more attention.^16^^,^^17^ When the intensity of the pulsed laser is above the plasma formation threshold, the plasma is generated in the irradiated sample and can be detected by an external circuit. The positive and negative ions in the plasma move directly under the external electric field and are collected and transmitted to an external circuit. Thus, a voltage signal is detected by the receiver of the measurement circuit and is termed as laser-induced voltage (LIV), which may be an effective means to characterize the plasma change process. LIV, sensitive to sample composition and structure, is a real-time method to be applied for characterizing materials and monitoring reaction processes with high sensitivity and experimental simplicity, and also for reflecting laser parameters in real time.^18^^,^^19^^,^^20^^,^^21^ It has been also used in food, oil, and gas resources testing and analysis.^22^^,^^23^^,^^24^^,^^25^^,^^26^

Table salt (TS) as a white single crystal is found in almost every household throughout the world. The main component of TS is NaCl, and it has the advantage of cheap, universally available. NaCl, only known stable compound in the Na-Cl system under ambient conditions,^27^ has many natural defects in TS such as vacancies, steps, and lattice distortions.^28^ As we know, NaCl is wide bandgap, ionic materials. It is well characterized by the interaction with ultraviolet (UV) photons because of defects.^29^ The NaCl-laser interaction has been an attractive research focus in many fields such as atmospheric science, environmental contamination, and pharmaceuticals.^30^^,^^31^^,^^32^ For this purpose, investigation of LIV from NaCl is of significance, especially for TS which is rich in defects.

Krypton fluoride (KrF) excimer laser has been widely used in industrial fields and scientific research because of its inherent smooth beam profile, short wavelength, high energy, narrow pulse width and high power, including semiconductor lithography, etching of hard or chemically stable materials, and research on inertial confinement fusion.^33^^,^^34^^,^^35^^,^^36^ Due to a high energy fluence the KrF pulsed laser generates a large amount of plasma when it is focused on the target. Therefore, KrF laser was selected as the irradiation laser source in this work. Based on the interaction between laser and TS, the LIV response reflects the plasma characteristics and motion processes. In the meantime, a function was established between the LIV amplitude (V_p_) and laser energy through the characteristics of LIP. Here, we also focus on the impedance effect of the LIV response, where the LIV response time was significantly reduced as the external resistance decreased. The research results showed that with a small external resistance, ln(V_p_) is linearly correlated with the incident laser energy and the LIV response time matches the laser pulse duration, so TS is expected to be a fast, easy-to-acquire, and low-cost material for detecting UV single-pulse laser.

## Results and discussion

**Figure 1:**
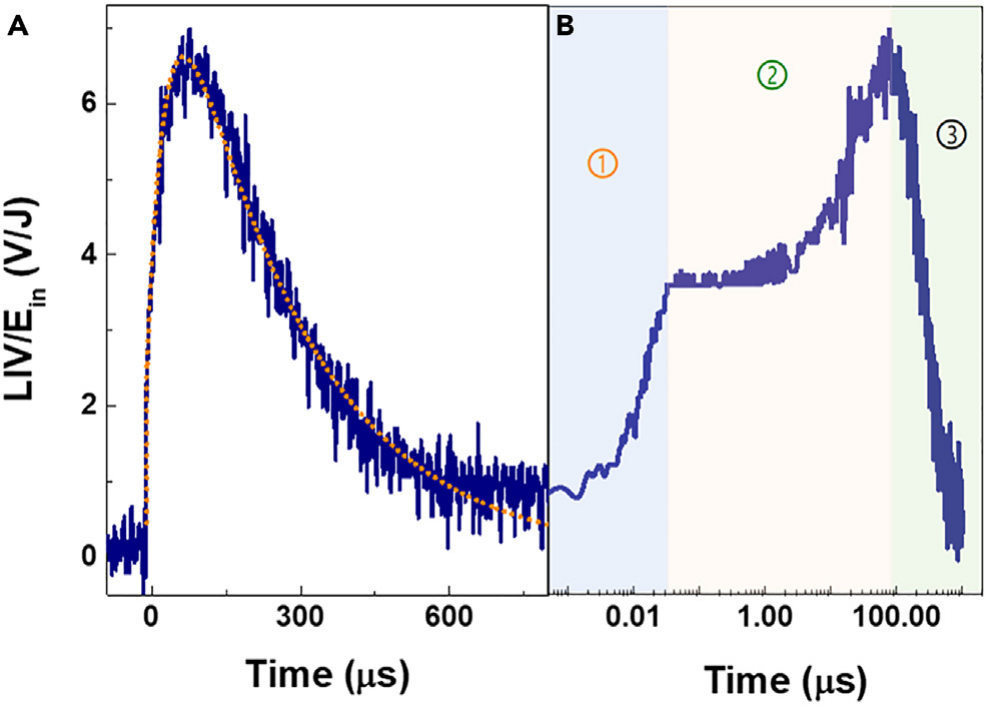
Time evolution of a typical LIV response of TS under a selected E_in_ of ∼20 mJ, V_b_ = 200 V, and R = ∞ (A) Linear axis, and dotted lines show the calculations with the three exponential functions. (B) Logarithmic axis.

If plasma is emitted from the TS surface, the LIV signal detected with the copper electrodes. Figure 1A shows the typical LIV signal detected using the oscilloscope when the KrF laser irradiated on the TS at V_b_ = 200 V, E_in_ ≈ 20 mJ, and R = ∞. The LIV response shows three variation processes: quick increase before 33 ns, a peak at ∼ 65 μs, and a gradual decay lasting ∼560 μs (Figure 1B). The time of plasma flight can often be modeled by a delta function source of effusing particles with a Maxwell-Boltzmann velocity distribution.^37^ Here, the data were well fitted by the sum of the three exponential functions with the decay times τ_1_ ∼ 12.5 ns, τ_2_ ∼ 30 μs, and τ_3_ ∼ 210 μs, LIV (t)/E_in_ = – A_1_e^-t/τ1^ – A_2_e^-t/τ2^ + A_3_e^-t/τ3^.^38^ The close correspondence of measured (solid blue lines) and the calculated traces (dashed orange line) indicated that the model is suited to describe the temporal development of the LIV response, where A_1_ = 5.97 V/J, A_2_ = 13.43 V/J, and A_3_ = 18.66 V/J (Figure 1A). This LIV response is divided into three parts: expansion, mobilization, and compounding processes of the plasma. A_3_ represents the weaker process of the plasma, so it has a positive parameter which is different from A_1_ and A_2_.

**Figure 2:**
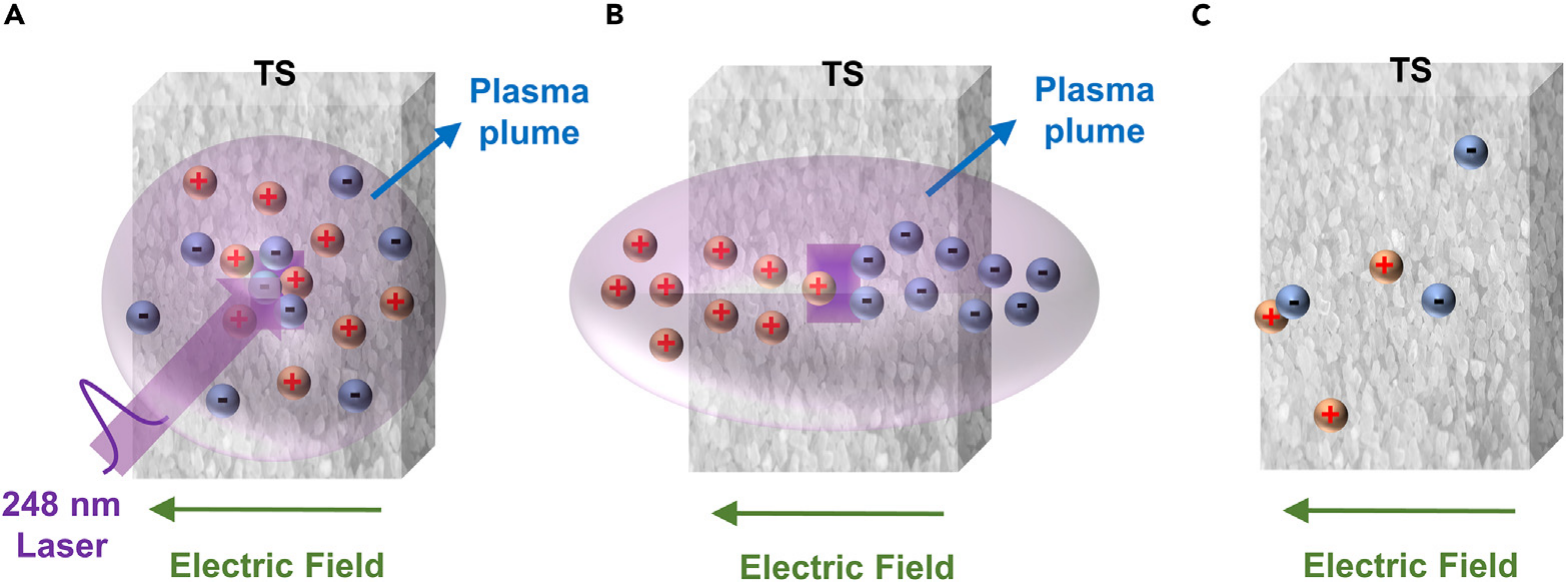
Dynamics of laser-induced plasma (A) The stage of free expansion. (B) The process of ambipolar diffusion under electric field. (C) The last stage of plasma in motion.

For nanosecond (ns)-pulsed laser irradiation of the TS powder, if the laser intensity is sufficiently large, the laser irradiation could form a plasma plume at the surface of the TS, whose generation and motion can greatly affect the LIV response. Thus, by measuring the induced voltage, we can understand more information about the produced plasma situation. The plasma consists of two main groups: a “fast” one composed of ionized species and a “slow” one which is mainly due to the contribution of neutral particles.^39^ The laser-induced plasma has the shape of semi-spherical three-dimensional symmetries under the ionization/recombination and excitation/de-excitation mechanism.^40^ At the initial stage of laser irradiation on the salt, due to shielding effects, main part of the laser energy E_in_ passes through inverse bremsstrahlung and partial reflection of the laser beam to heat the electrons instead of coupling directly to the sample.^41^ During laser irradiation of 20 ns the plasma is in thermal equilibrium. But after pulse termination, the cloud enters a non-equilibrium state and expands to the ambient gas at pressure P_c_ (P_c_ > P_0_, P_0_ is the natural environmental pressure).^42^ Here, the plasma is ejected in the very strong field left by the laser excitation through a Coulomb process in a very short timescale by laser excitation. In this initial stage plasmas are very hot and dense, which can be approximated by a free expansion. The plasma expanding to the vicinity of the electrodes is captured by an oscilloscope and converted to a LIV signal (Figure 2A).

The subsequent slow-rising process needs more time to establish, because the neutral particles in the subsequent thermal process are influenced by the V_b_.^43^ In this work, plasma occurs the ambipolar diffusion in the cuvette due to the electric field. The electrons have higher mobility and early spread to the electrodes. The positive charge leaving behind will retard the motion of electrons and speed up the ions.^44^ The plasma expansion can be weakened by plume confinement, collisions between plasmas and scattering effects in atmospheric environments. The applied electric field V_b_ can accelerate the process of ambipolar diffusion and reduce the loss of plasma in motion (Figure 2B). When the plasma reaches gas-dynamic equilibrium, the quartz cuvette still contains electrons number density of 10^16^-10^19^ cm^−3^.^45^ The plasma moved to the electrodes under the force of external electric field and then converted into an electrical signal. In addition, the existence time of this process is also affected by the collisions between different types of plasma particles, and the complex space distribution of the electrostatic retarding field and the shielding effect. The LIV signal decreases sharply with laser shot following the gradual waning of the plasma, and eventually returns to its initial state (Figure 2C).

**Figure 3:**
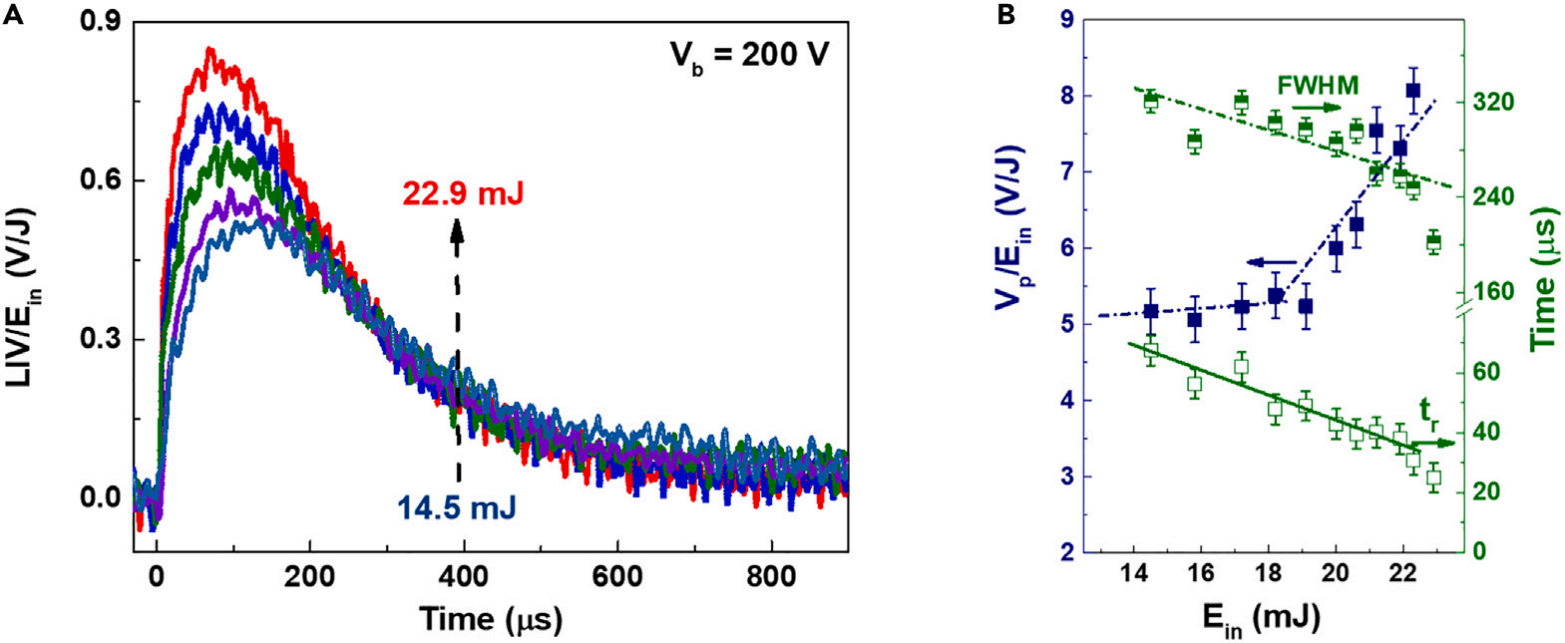
LIV response from table salt with different E_in_ at V_b_ = 200 V, which were measured without load resistance (A) The LIV traces. (B) V_p_/E_in_ (solid point), FWHM (half-up point), and rise time t_r_ (open point) as a function of E_in_. Data are represented as mean ± SEM. V_p_/E_in_’s SEM = 0.3 V/J, FWHM’s SEM = 10 μs, and rise time t_r_’s SEM = 5 μs.

To illustrate the effect of laser energy on the LIV traces characteristic, the change of the LIV signals with the laser energies are shown in Figure 3A. In order to further determine the relationship between the LIV signal and the laser energy E_in_, we extracted the values of a maximum voltage V_p_, an l0%–90% rise time t_r_ and a full width at half-maximum (FWHM) of LIV traces. The results are shown in Figure 3B, where V_p_/E_in_ increases with increasing E_in_, while FWHM and t_r_ decrease almost linearly. This LIV signal is believed to originate from the combined action of the plasma generated by laser irradiation and the electric lines of forces distributed in the space around the electrode due to the applied V_b_. At high E_in_ the number of plasmas increase, thereby increasing the LIV response. In the same way, when the laser energy increases, the sample surface creates a larger lateral pressure gradient and temperature gradient, accelerating the sprayed particles and “pushing” them to electrodes. Thus, the oscilloscope detects the plasma earlier and the FWHM and t_r_ decrease with increasing E_in_. The slope of FWHM and rise time t_r_ against E_in_ is −10.38 and −4.48 μs/mJ. Here, the responsivity R∗ is defined as R∗ = V_p_/E_in_. Therefore, the experimental setup has an R∗ of ∼5.22 V/J when E_in_ < 19.1 mJ and a maximum R∗ of 8.07 V/J at E_in_ = 22.3 mJ.

**Figure 4:**
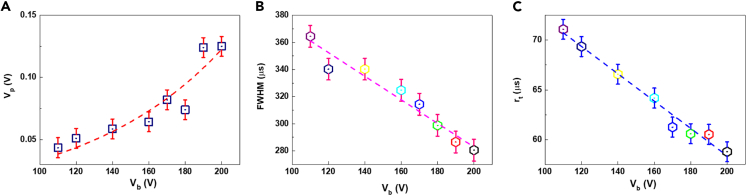
LIV signal parameters of salt as function of V_b_ when E_in_ = 18.2 mJ (A) The relationship between V_p_ and V_b_. Data are represented as mean ±0.008 V. (B) The relationship between FWHM and V_b_. Data are represented as mean ±8 μs. (C) The relationship between R_t_ and V_b_. Data are represented as mean ±1 μs.

In Figure 4A, with the supplied voltage V_b_ is increased further, the V_p_ increases proportionally under a laser energy E_in_ of 18.2 mJ with R = ∞. The V_p_ increases from 43.5 to 125 mV with V_b_ from 110 to 200 V, and the relationship between V_p_ and V_b_ is described as V_p_ = ae^bVb^ with a = 9.3 mV and b = 12.8 V^−1^. It can be seen in Figures 4B and 4C that the FWHM and r_t_ strongly depend on the applied voltage and decrease linearly with the V _b_. FWHM and t_r_ as the function of V_b_ are presented as FWHM = α_1_V_b_ + β_1_ and t_r_ = α_2_V_b_ + β_2_, where α_1_ = −0.87 μs·V^−1^ and β_1_ = 456.8 μs, α_2_ = −0.137 μs·V^−1^ and β_2_ = 85.8 μs. With this electrode configuration, a portion of the electric lines can interact with the plasma due to the horizontally electric field, promoting the movement of the plasma toward the electrode and resulting in a LIV signal. When the V_b_ increases, the plasma expanding speed is faster and faster, so the plasma reaches the electrodes very early, and FWHM and t_r_ decrease with the V_b_. Meanwhile, a larger V_b_ leads to a stronger electric field effect, which increases the LIV signal amplitude.

**Figure 5:**
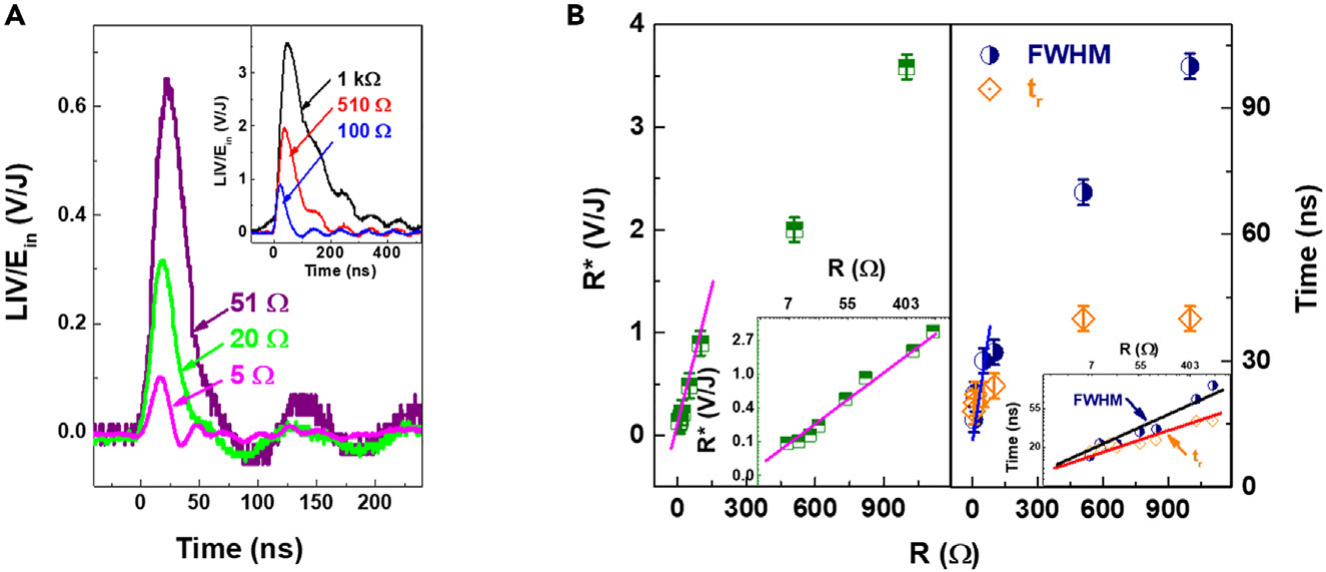
Temporal evolution of the LIV signals below 1 kΩ with a selected E_in_ of ∼14 mJ and V_b_ = 200 V (A) The LIV traces. (B) R∗, FWHM and rise time t_r_ as a function of R. Data are represented as mean ±0.12 V/J, ±3 ns, ±3 ns, respectively.

It is shown that the V_p_ of the LIV response characterizes the plasma density. The pulse width of KrF laser is 20 ns, but the response time of LIV is on the ms scale. In addition to characterize the dynamical processes of the plasma, we consider that the LIV signal response is affected by the impedance of the test circuit. Figure 5A displays the LIV waveforms with different external resistors R at V_b_ = 200 V and E_in_ = 14.5 mJ. The LIV signal amplitude V_p_ decreased from 3.58 mV to 0.1 mV when the R changed from 1 kΩ to 5 Ω. These waveforms indicate that the LIV response process is determined by line resistance. In order to analyze the LIV response characteristics, Figure 5B provides an insight study of the FWHM and t_r_ at different R. The responsivity R∗ as a function of R is shown in Figure 5B and a simple form of R∗, FWHM and rising time t_r_ is given for R < 100 Ω, where R∗∝ R, FWHM ∝ R and t_r_ ∝ R. A careful simulation conforms to the form of ln (R∗) = kln(R), where k ≈ 0.49 ln(V/J)/ln(Ω). Both the R∗, FWHM and rise time r_t_ show high values of ∼3.59 V/J, 100 ns, and 40 ns at R = 1 kΩ and decrease gradually to ∼0.13 V/J, 16 ns and 18 ns at R = 6.7 Ω. The ln (FWHM) and ln (t_r_) against lnR display approximate slopes of ∼0.34 and 0.16 ln(ns)/ln(Ω) (insets of Figure 5B). Here, the LIV response is mainly influenced by impedance effects.

**Figure 6:**
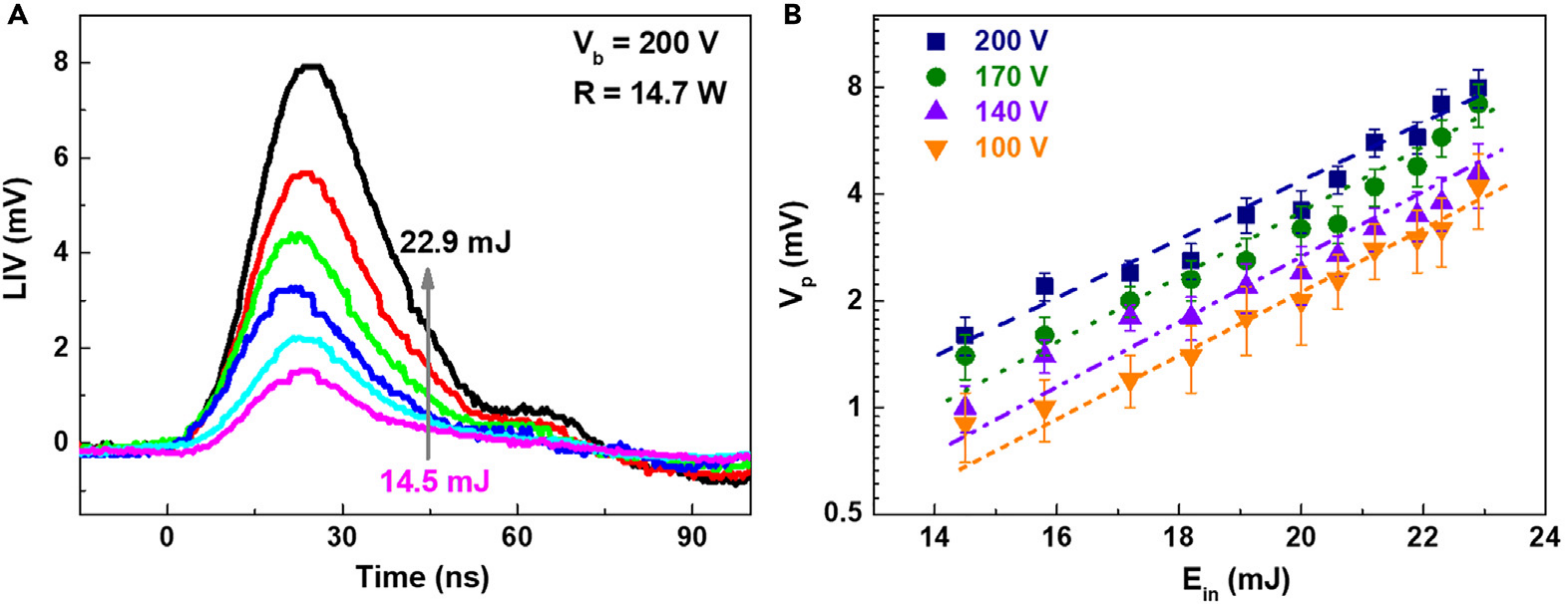
LIV as a function of E_in_ under and R = 14.7 Ω (A) The LIV traces when V_b_ = 200 V. (B) V_p_ as a function of E_in_ selected V_b_ of 200, 170, 140 V and 100 V. Error bars represent standard deviation, with the fluctuations of ±9%, ±12%, ±11% and ±20%, respectively.

The response time of LIV signal can be reduced to ∼20 ns, which is equal to the pulse width of the KrF pulsed laser, by adjusting the load resistance R in the circuit. R rapid discharge leads to LIV response enhancement, but with loss of plasma dynamics process information. The results in Figure 5 display that the FWHM ∼21 ns and the rise time t_r_ ∼ 22 ns of LIV signal when R = 14.7 Ω. The response characteristics are similar to the KrF pulsed laser. Figure 6A shows the typical LIV transients to pulsed laser irradiation with different E_in_ under V_b_ = 200 V and R = 14.7 Ω. These LIV responses show similarly triangular and symmetrical traces, and the time constant is completely limited by the laser pulse duration. Consistent with the findings in Figure 3B, the LIV signal peak V_p_ is elevated with increasing laser energy E_in_ as shown in Figure 6B, satisfying the logarithmic relationship of ln(V_p_) ∝ E_in_ with a slop of ∼0.17 ln(mV)/mJ, slightly V_b_ dependent. Meanwhile, this result provides a possible way for real-time detection of laser pulse characteristics.

**Figure 7:**
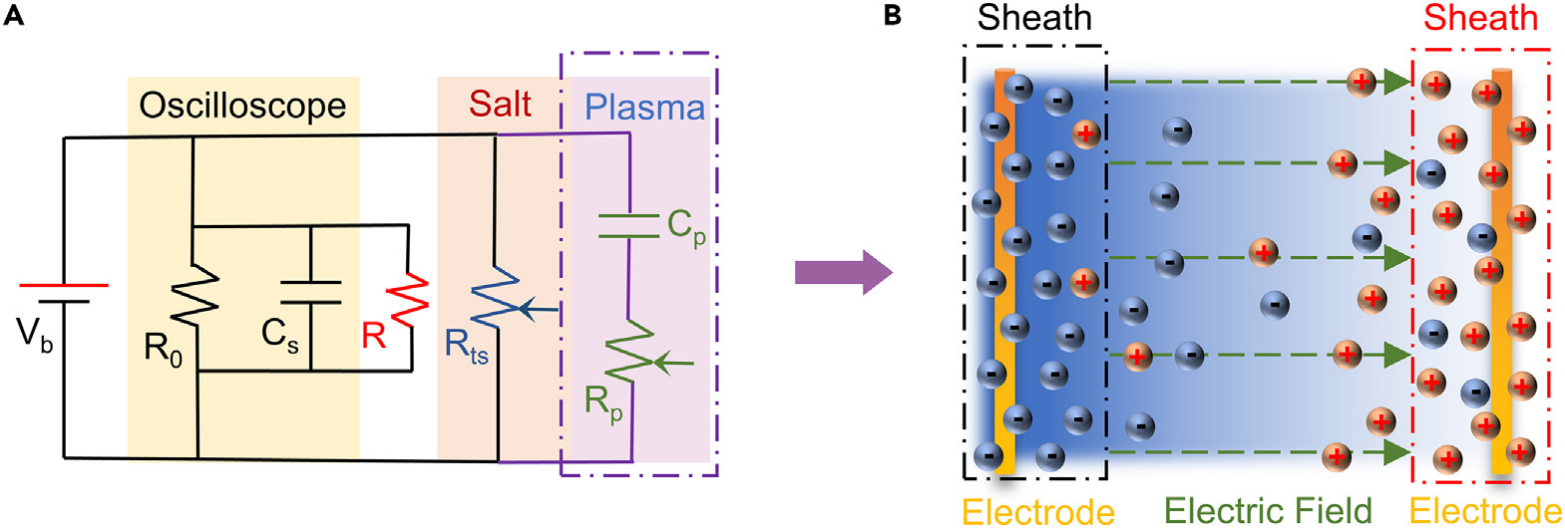
The diagram of measurement (A) The equivalent circuit. (B) The plasma diffusion.

The oscilloscope can be equated to a resistor and capacitor in parallel, so the response time of the circuit is affected by the resistor-capacitance circuit constant, and a lower R will result in a much shorter decay time of the LIV response. The periodic oscillation of ∼20 ns at the end of LIV trace, which may be due to an impedance mismatch in the circuit caused by the mismatch between the co-axis cable and the 1 MΩ oscilloscope. The impedance change detected by the circuit throughout the detection process is composed of the structural impedance and the plasma impedance generated by the sample (Figure 7A). Considering the test circuit as a voltage divider, the real signal U_ts_ generated by TS under the laser irradiation is calculated from the oscilloscope readout voltage U_o_ by U_ts_ = U_o_(1+R_ts_/R_i_), where R_ts_ is the sample impedance and R_i_ = R//R_0_ ≈ R is the impedance of the oscilloscope input channel. Here the LIV signal amplitude is mainly dependent on the circuit impedance, so the V_p_ increases monotonically with the impedance R.

The plasma is emitted from the TS because of laser irradiation. At the same time, heating, melting, boiling, evaporation, and phase explosion occur on the surface of TS.^46^^,^^47^ In fact, laser induced plasma contains many charged particles including electrons and ions, which can be driven by an electric field. Here, the laser induced plasmas can be considered as an equivalent circuit as shown in Figure 7A. Due to the generalized flow is driven by different kinds of generalized forces existing in the system, the plasma is driven by density gradient and temperature gradient to the vicinity of the electrodes for rapid expansion. At this moment, the plasma density and temperature are highest in the center of pulsed laser irradiation. The temperature of the plasma increases after absorbing laser energy and gradually decreases after expanding outward. The supplied electric field cannot penetrate the region of high density plasma aggregation, meaning that the process is not affected by V_b_. The electrons in TS can be easily excited to air due to the low electron affinity of NaCl. Since the mass of positive ions are much larger than electrons, the speed of electrons is much larger than that of ions. Therefore, throughout the diffusion process, electrons will collide with the electrodes earlier than the ions and accumulate near the electrodes.

As shown in Figure 7B, electrons and positive ions are collected near the electrodes on both sides by diffusion and external electric field. Here, we focus on the process of electron movement. The electrons will prevent electrons from moving near the electrode after reaching a certain amount. Simultaneously, some positive ions move toward the electrode where the electrons accumulate, and neutralize some electrons under the effect of density gradient, resulting that the ability to stop electrons is weakened. Finally, the two kinds of particles achieve dynamic equilibrium near the electrodes, and this small area is defined as the sheath. Thus, the rising part of LIV signal is divided into two phases due to the change of the plasma dynamics process. And because of the sheath, the LIV signal is influenced by the incident laser energy and bias voltage.

In this work, the laser energy changes are reflected by the kinetic characteristics of LIP, unlike conventional photoelectric effect. We investigated the characteristics of TS on deep UV single-pulsed laser detection through LIV signals. The LIV responses are positively correlated with the laser energy. The response time of this measurement system is reduced to ∼20 ns by changing small external resistor, which provides a prerequisite for the application of single-pulse laser energy detection. The direct detection of LIP kinetic processes in real time is an important practical problem. The LIV method opens up new possibilities for kinetic research on laser-induced plasma, exhibiting a performance level and application promise. We accurately monitored three processes in the LIP of TS. The LIV method holds potential to improve the LIP process monitoring and, combining with micro-drilling machining, will improve the efficiencies of process.

Laser-induced plasma is a very complex and various phenomenon as it depends on laser pulse characteristics, as well as on the state of the irradiated sample and its chemical and physical properties. The dynamics of laser-induced plasma are highly dependent on the environment where plasma generated, such as vacuum, atmospheric, and gaseous background, meanwhile, the energy exchange at the contact wall is formed, which plays a crucial role on the expansion dynamics.^48^ The background environment of present research for LIV was atmospheric, and the LIV signal of TS under laser irradiation with different pulsed energy E_in_ differs significantly because of the motion properties of the plasma. The corresponding relation between laser parameters and electrical signals was found by analyzing the characteristics of the LIV. The effect of electrode geometry on spark ignition has been investigated in recent years, but most of the researches have focused on the theoretical modeling stage. It would be very meaningful and interesting to observe the plasma dynamics when the pressure in the quartz cuvette is reduced down to a reasonable vacuum (1 × 10^−5^ mbar). It could be also interesting for modifying the electrodes geometry and the material in the cuvette to obtain a more complete characterization of the plasma dynamics.

### Conclusion

In summary, the LIV response of TS combined with the impedance effect was used to achieve rapid detection of single-pulse UV laser. The entire LIV response consists of three parts of fast rise, slow rise, and slow fall, which is influenced by the plasma motion process. The V_p_, FWHM, and r_t_ of the LIV signal were affected by V_b_, E_in,_ and R. The impedance of the entire test circuit was matched to the oscilloscope when R = 14.7 Ω, resulting in the LIV response time equal to the laser pulse width (≈20 ns), the responsivity R∗ of ∼0.17 V/J, and the ln(V_p_) linearly dependent on E_in_ with a slope of ∼0.17 ln(mV)/mJ. We have displayed a method detecting and analyzing laser-matter interactions while also enabling real-time detection of the single-pulse laser. This work greatly simplifies the process of converting the LIV signal monitored by the circuit into laser energy, and provides a new insight of simple, low-cost, ultra-fast response for ns pulse laser characterization.

### Limitations of the study

In recent years, ultra-short-pulse laser technologies developed rapidly and demonstrated their research and application value in multiple aspects. This work only focused on the characteristics of LIV under a ns laser irradiation and the response time limit of TS has not been confirmed yet. Further experimental research is needed to investigate the LIV response of salt under picosecond (ps) or femtosecond (fs) laser irradiation. In addition, due to the voltage precision of ∼0.1 mV for an oscilloscope, an applied bias of V_b_ higher than 100 V was used in this work to ensure the high signal-to-noise ratio of LIV response. In order to meet the industrial demand for lower-power consumption in measurement, single pulse laser detection based on TS could be improved to operate at a low bias in future experiments.

## STAR★Methods

### Key resources table

**Table:** 

REAGENT or RESOURCE	SOURCE	IDENTIFIER
**Chemicals, peptides, and recombinant proteins**		

Table Salt	China National Salt Industry Group Co., Ltd	http://gufen.chinasalt.com.cn/zy/Product/A006002Gone1.html

### Resource availability

#### Lead contact

Further information and requests for resources and reagents should be directed to and will be fulfilled by the Lead Contact, Kun Zhao (zhk@cup.edu.cn).

#### Materials availability

This study did not generate new unique reagents.

#### Data and code availability

• All data reported in this paper will be shared by the lead contact upon request.
• This paper does not report original code.
• Any additional information required to reanalyze the data reported in this paper is available from the lead contact upon request.

### Experimental model and study participant details

Our study does not use experimental models typical in the life sciences.

### Method details

Figure S1 shows the measurement system employed in this work. A KrF excimer laser COMPexPro50 from Coherent Inc. was used as the source at room temperature in air, operating at a wavelength of 248 nm (5 eV photon energy) with 20 ns duration at a 1 Hz repetition rate in this experiment. The maximum pulse energy of laser is 150 mJ with the maximum pulse frequency of 50 Hz and the average power of 7 W. The beam dimensions and the beam divergence are 14 × 7.5 mm^2^ and 2 × 1 mrad,^2^ respectively. At the distance of 90 mm from the sample, the laser irradiated vertically onto the sample surface through a 4 mm × 7.5 mm diaphragm. The pulse energy E_in_ on sample was varied between 13 and 25 mJ. The sample was filled into a quartz cuvette with geometry of 20 mm × 45 mm and a thickness of 10 mm. Four parallel copper electrodes with 2 mm intervals were fixed in the cuvette. In the above configuration, the two electrodes near the side wall of the cuvette were supplied with a DC voltage V_b_. Here, the power supply was derived from the Keithley 2400 source meter. The other two adjacent electrodes were connected to an adjustable resistor R and an oscilloscope. In order to reduce the response time of the detected signal, R was changed from 1 kΩ to 5 Ω. The LIV trace was measured through the Tektronix DPO4032 sampling oscilloscope (350 MHz bandwidth terminated into R_0_ = 1 MΩ and 2.5 GS/s sampling rate) with the LIV signal as an input to signal port of the oscilloscope, and the oscilloscope is grounding during signal detection. TS was filled into the cuvette at a high of 21.5 mm. The air pressure in the cuvette is 101 kPa (P_0_) which corresponds to the outside environment.

TS in present experiment was from China National Salt Industry Group Co., Ltd. As-supplied TS powder without any processing has been directly placed on the sieve. TS particles were fractionated by manual shaking for approximately three minutes using four sieves with mesh sizes of 550, 380, 250 and 180 μm, thus particle size classes were indicated with the corresponding size range of > 550 μm, 380-550 μm, 250-380 μm, 180-250 μm and < 180 μm. The samples were screened for five times to ensure the size distribution of 7.1%, 21.8%, 46%, 15% and 10.1% particles are < 180 μm, 180-250 μm, 250-380 μm, 380-550 μm and > 550 μm, respectively. In order to measure accurately, the data was the mean value of the five pulse tests. In this study, the component information of the TS was examined with X-ray diffraction (XRD). As shown in Figure S2, the XRD peaks of (111), (200), (220), (222), (400), (420), and (422) indicated that the main component of TS was cubic phase NaCl.

### Quantification and statistical analysis

The Origin 9 package was used for statistical analysis. Data reported are expressed as the mean of five separate laser pulse experiments. SEM was used for error bars.
