# Supplementary material for: Laser-induced voltage of table salt for deep ultraviolet pulsed laser detection
Source: iScience. 2024 Mar 5;27(4):109424. doi: 10.1016/j.isci.2024.109424 (PMC10952038; doi:10.1016/j.isci.2024.109424)
Supplement: Document S1. Figures S1 and S2 [file mmc1.pdf]

iScience, Volume *27*

Supplemental information

Laser-induced voltage of table salt for deep ultraviolet pulsed laser detection

Xuecong Liu, Kun Zhao, and Xinyang Miao

Supplemental information

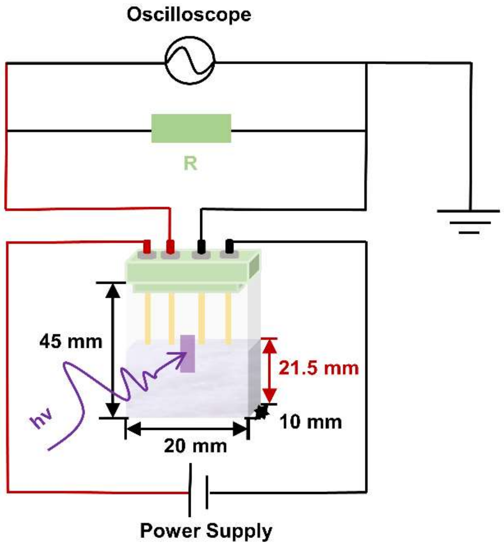

Figure S1. Schematic diagram of the measurement system. Related to EXPERIMENTAL.

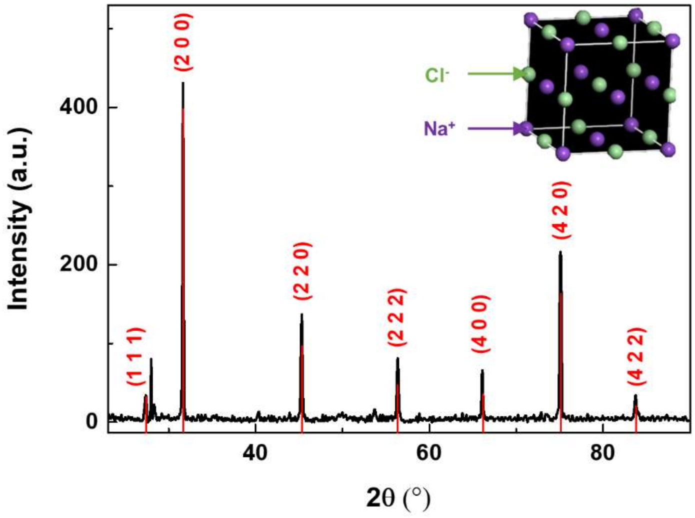

Figure S2. XRD patterns of TS. Inset is the crystal structure of NaCl.

Related to EXPERIMENTAL.
